# Mitochondrial dysfunction in Spaceflight Associated Neuro-Ocular Syndrome (SANS): a molecular hypothesis in pathogenesis

**DOI:** 10.1038/s41433-024-02951-3

**Published:** 2024-02-07

**Authors:** Ethan Waisberg, Joshua Ong, Mouayad Masalkhi, Xiao W. Mao, Afshin Beheshti, Andrew G. Lee

**Affiliations:** 1 Department of Ophthalmology, University of Cambridge, Cambridge, UK; 2 Department of Ophthalmology and Visual Sciences, University of Michigan Kellogg Eye Center, Ann Arbor, MI USA; 3 School of Medicine, University College Dublin, Belfield, Dublin Ireland; 4 Division of Biomedical Engineering Sciences, Department of Basic Sciences, Loma Linda University Health, Loma Linda, CA USA; 5 Blue Marble Space Institute of Science, Space Biosciences Division, NASA Ames Research Center, Moffett Field, CA USA; 6 Stanley Center for Psychiatric Research, Broad Institute of MIT and Harvard, Cambridge, MA USA; 7 Center for Space Medicine, Baylor College of Medicine, Houston, TX USA; 8 Department of Ophthalmology, Blanton Eye Institute, Houston Methodist Hospital, Houston, TX USA; 9 The Houston Methodist Research Institute, Houston Methodist Hospital, Houston, TX USA; 10 Departments of Ophthalmology, Neurology, and Neurosurgery, Weill Cornell Medicine, New York, NY USA; 11 Department of Ophthalmology, University of Texas Medical Branch, Galveston, TX USA; 12 University of Texas MD Anderson Cancer Center, Houston, TX USA; 13 Texas A&M College of Medicine, Texas, TX USA; 14 Department of Ophthalmology, The University of Iowa Hospitals and Clinics, Iowa City, IA USA

**Keywords:** Pathogenesis, Diseases

## Introduction

Spaceflight-associated neuro-ocular syndrome (SANS) is a condition affecting astronauts during long-duration spaceflight (LDSF). SANS is characterized by hyperopic refractive shifts [1], optic disc edema [2], globe flattening, and chorioretinal folds [3]. With the commercialization of human spaceflight, the amount of space travelers experiencing LDSF will grow exponentially over the coming years, and there may be a potential increase in cases of SANS. In addition, the planned crewed 2030 Mars Mission will expose astronauts to LDSF greater than previously experienced [4]. Although, the exact mechanisms underlying SANS is not fully understood, it is believed to be related to the unique environment of LDSF [5]. SANS has been traditionally linked to fluid shifts and structural changes in the eye caused by microgravity [5]. This hypothesis is based on that the absence of gravity in space leads to a redistribution of bodily fluids, resulting in increased intracranial pressure and subsequent changes in the eye [5]. However, recent research is exploring the role of mitochondrial dysfunction as a possible underlying cause of SANS [6].

Microgravity and increased exposure to galactic cosmic radiation during spaceflight are known to have significant impacts on mitochondrial function [7]. Recent multi-omics and systems biology analysis of data from 59 astronauts and mice and data from NASA’s GeneLab found that mitochondrial stress is a consistent phenotype of spaceflight [8]. This remarkable study highlights how spaceflight significantly impacts mitochondrial function, resulting in altered gene expression, reduced antioxidant defences, and increased oxidative stress [8]. These changes affect metabolic pathways and gene expression, indicating that mitochondrial dysfunction is a critical consequence of long-duration space missions and could potentially be connected to SANS development [8].

Mitochondria are essential cellular organelles responsible for energy production through oxidative phosphorylation [7]. They play a crucial role in regulating apoptosis and maintaining cellular homeostasis, neuronal excitability and synaptic transmission [7]. Mitochondria play an essential role in the eye, aiding with the transmission of visual information from the retina to the brain, and are densely concentrated in the retinal ganglion cell axons [9]. The inner segments of retinal photoreceptors have an abundance of mitochondria, allowing for outer segment renewal and phagocytosis [10]. The retina ages significantly faster than other organs due to its elevated density of photoreceptors, and an individual experiences a decline of 70% in ATP production throughout their lifetime [11].

Diabetic retinopathy and Age-related macular degeneration are two major eye conditions associated with mitochondrial defects [12]. The retina is highly susceptible to oxidative damage due to its high oxygen consumption and exposure to light [12]. Excessive production of reactive oxygen species (ROS) damages retinal cells and contributes to disease progression [12]. Leber hereditary optic neuropathy is another critical example of a mitochondrial disease leading to vision loss, characterized by acute or subacute bilateral loss of central vision [13]. This diversity in diseases underlines the crucial role of mitochondria in maintaining retinal health.

Microgravity conditions can alter cellular signaling pathways, leading to mitochondrial dysfunction and subsequent cellular damage [14]. Recent research has also shown that other effects of microgravity on mitochondrial function include alterations in gene regulation, lipid metabolism and innate immunity [8]. Microgravity is also associated with decreased metabolic demands which may reduce the expression of proteins involved in oxidative phosphorylation [6].

During LDSF, astronauts are outside of Earth’s protective magnetosphere, which exposes the eye to significantly higher levels of ionizing radiation, which has known deleterious effects on mitochondria [15, 16]. Additionally, the space environment exposes astronauts to increased levels of oxidative stress, which can further compromise mitochondrial function [17]. Oxidative stress occurs when there is an imbalance between the production of ROS and the ability of cellular antioxidants to neutralize them [17]. Mitochondria are both sources and targets of ROS, and their dysfunction can potentially contribute to the oxidative stress observed in SANS [17].

Mao et al. previously showed in a rodent study that spaceflight conditions induced mitochondrial oxidative damage in ocular tissue [18]. Levels of 4-hydroxynonenal (4-HNE) protein, a specific marker for lipid peroxidation was significantly elevated in the retina of mice following spaceflight compared to ground-control mice, and significant apoptosis was seen in the inner nuclear layer and ganglion cell layer of the retina in the spaceflight mice [18]. Additional research will be required to examine the effects of spaceflight on mitochondrial function in-vivo with retinal flavoprotein autofluorescence imaging, which can non-invasively assess retinal dysfunction prior to the death of retinal cells.

In a study of 49 astronauts by Zwart et al. [19], B-vitamin status was shown to be a significant predictor of visual outcomes observed after spaceflight (*P* < 0.001).

B-vitamin are essential nutrients, that are known to directly regulate the metabolism of mitochondria [20]. Vitamin B3, nicotinamide, is known to promote retinal pigment epithelium differentiation and enhance mitochondrial metabolism. This same study by Zwart et al. also found that affected astronauts had higher levels of 1-carbon metabolites (such as homocysteine) prior to spaceflight [19]. Homocysteine is known to affect mitochondrial function, structure and energy production, and has been shown to have neurotoxic effects on ischemic brain cells [21]. An excess of 1-carbon metabolites occurring in astronauts during LDSF may lead to mitochondrial dysfunction, which potentially manifests as SANS in the eye.

Understanding the precise mechanisms linking mitochondrial dysfunction to SANS is crucial for developing effective countermeasures to mitigate the risks associated with long-duration space missions. These countermeasures may include lifestyle and pharmacological interventions and modifications aimed at preserving mitochondrial health. Emerging countermeasures for SANS include the use of swimming goggles to provide a moderate increase in intraocular pressure to mitigate SANS [22], and the use of augmented reality to restore any potential losses in visual function [23, 24]. Near-infrared light therapy may also be a useful side effect free, and non-invasive method to improve mitochondrial function during spaceflight. Improved diagnostics methods in space will also be essential as well to detect subtle structural changes during spaceflight [25, 26]. Further research will be required to determine the effects of mitochondrial function on the development of SANS and the efficacy of various countermeasures.
